# A rare homozygous missense mutation of *COL7A1* in a Vietnamese family

**DOI:** 10.1038/s41439-022-00192-y

**Published:** 2022-05-17

**Authors:** Nguyen Thuy Duong, Luong Thi Lan Anh, Nguyen Huu Sau, Nguyen Bao Anh, Noriko Miyake, Nong Van Hai, Naomichi Matsumoto

**Affiliations:** 1 Institute of Genome Research, Vietnam Academy of Science and Technology, Hanoi, Vietnam; 2 Hanoi Medical University Hospital, Hanoi Medical University, Ministry of Health, Hanoi, Vietnam; 3 Hanoi Medical University and National Hospital of Dermatology, Ministry of Health, Hanoi, Vietnam, Hanoi, Vietnam; 4 National Research Institute, National Center for Global Health and Medicine, Tokyo, Japan; 5 Yokohama City University Graduate School of Medicine, Yokohama, Japan

**Keywords:** Genetics, Molecular biology

## Abstract

We present a homozygous missense mutation in the *COL7A1* gene (NM_000094.4: c.6262G>A, p.G2088R) in a case of inversa recessive dystrophic epidermolysis bullosa (RDEB-I) from a nonconsanguineous Vietnamese family. Although a heterozygous form of this mutation in combination with a premature termination codon allele has been shown to cause RDEB-I, this is the first report of homozygosity of this mutation as the etiology. Here, we investigated the molecular basis of the patient’s disease for prenatal diagnosis after genetic counseling of the parents.

## Introduction

Epidermolysis bullosa (EB) is a group of disorders characterized by blisters and fragile skin formation. A decrease or lack of function of proteins involved in epidermal and dermal-epidermal adhesion in the skin basement membrane zone (BMZ) is found to be the cause of EB^1^. EB is often categorized into four major types, EB simplex, junctional EB, dystrophic EB, and Kindler’s syndrome, depending on the ultrastructural level of the BMZ in which the blisters form. Dystrophic EB mostly results from mutations in protein Col7^2,3^. RDEB-I is a rare, autosomal recessive subtype of dystrophic EB. Symptoms may include neonatal generalized blisters in early childhood that may heal over the years; development of lesions of the oral, esophageal, anal, or genital mucosa; and transition of blisters from extremities to flexures and the body axis as patients age^4,5^.

The *COL7A1* gene encoding Col7 is associated with RDEB-I. Col7 is the major component of the anchoring fibrils, attachment structures extending from the subbasal lamina of the dermoepidermal junction to the papillary dermis of the skin^6^. Col7 consists of three proα1 (VII) chains, each consisting of a central collagenous domain^6^. This is called the triple helix domain (THD), as it possesses Gly-X-Y patterned sequences that fold into a triple helix^4,6^. The THD contains 20 subdomains, numbered COL1 to COL20, with 19 interruptions in between, providing conformational flexibility to the THD^4,7^. Here, we present a case of a 9-year-old Vietnamese patient and his affected brother who expressed symptoms and molecular evidence of RDEB-I.

The study was approved by both the Institutional Review Board of the Institute of Genome Research, Vietnam Academy of Science and Technology, and the institutional review board of the Yokohama City University Faculty of Medicine. Informed consent was also obtained from the parents.

Genomic DNA was isolated from the whole blood of I.1, I.2, II.1, and II.2 and from amniotic fluid cells of II.3. Whole-exome sequencing (WES) was performed on proband II.1. The DNA library was prepared using the SureSelectXT Human All Exon 50 Mb, v5 libraries (Agilent Technologies, Santa Clara, CA, USA) and run on the 2500 platform (Illumina) with 101 bp paired-end reads (Illumina, USA). Quality-controlled reads were aligned to the human reference genome (UCSC hg19, NCBI build 37.1) using NovoAlign (http://www.novocraft.com/products/novoalign/). Polymerase chain reaction (PCR) duplications were removed using Picard (http://broadinstitute.github.io/picard/). Variants were called using the Genome Analysis Toolkit (GATK) (https://www.broadinstitute.org/gatk/index.php).

**Fig. 1:**
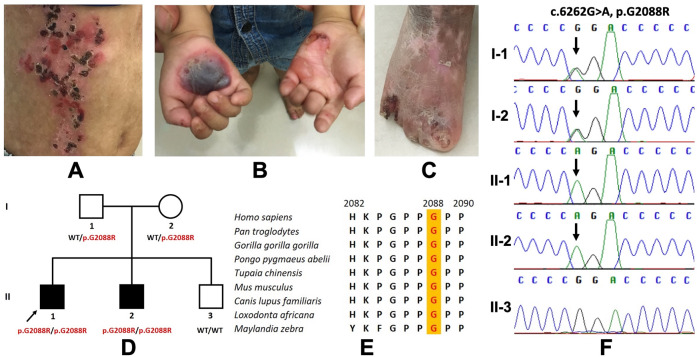
Proband at the age of 4 and the pedigree analysis of the family in the study. **A**–**C** Blisters and erosions on the trunk, palm creases, extremities and toes; **D** Family pedigree of the proband; **E** Multiple sequence alignment at amino acid position 2088 (highlighted in red); **F** Sanger sequencing results of the proband and his affected brother, healthy parents, and healthy brother.

The proband exhibited blisters and erosions on the trunk and extremities, dystrophy of the toes, palm creases, toe fusion, and enamel hypoplasia (Fig. 1A–C). WES showed a homozygous missense mutation of *COL7A1* (p.G2088R). Sanger results revealed that the proband’s affected brother was homozygous for the mutation, while his parents were heterozygous (p.G2088R) (Fig. 1D). Prenatal diagnosis for II.3 was performed using amniotic fluid cells, and a healthy brother was born with no pathogenic variants (Fig. 1F). Cluster Omega indicated the conservation of amino acid p.G2088 among humans and eight other species (Fig. 1E). The p.G2088R was predicted to be deleterious using in silico prediction tools (SIFT, Polyphen2, and MutationTaster).

**Fig. 2:**
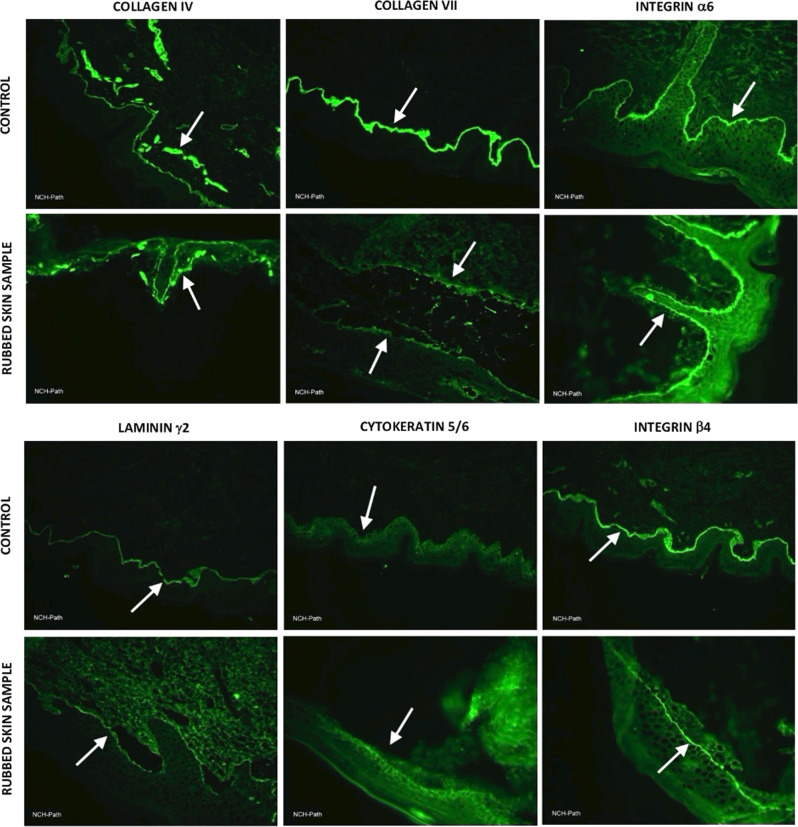
Immunofluorescence of rubbed skin samples from the proband’s forehead with corresponding healthy control samples. Immunofluorescence staining was performed with antibodies against collagen IV, collagen VII, integrin α6, integrin β4, laminin γ2, and cytokeratin 5/6. White arrows indicate the fluorescence signals of these proteins. The proband (rubbed skin sample) had a homozygous variant, p.G2088R, which was predicted to be deleterious by in silico prediction tools. Regarding collagen VII, while the control showed a broad and clear signal, the proband showed low and interrupted signals, indicating a significant reduction in collagen VII. However, the collagen IV signal of the proband was similar to that of the control. The proband sample presented a slight reduction in laminin γ2 and integrin β4, which are indicators for the diagnosis of junctional EB. The signals of integrin α6 and cytokeratin 5/6, which are indicators for the diagnosis of basal EB simplex and junctional EB, were similar to those of controls.

Immunofluorescence mapping (IFM) was performed using damaged tissue samples collected from the proband’s forehead (rubbed skin) with antibodies against collagen IV, collagen VII, integrin α6, integrin β4, laminin γ2, and cytokeratin 5/6. The proband’s rubbed skin showed a similar collagen IV staining fluorescence signal as the control but a remarkable loss of signal in collagen VII staining (Fig. 2). This observation, coupled with the genetic testing of the proband’s family and clinical symptoms, supports a diagnosis of RDEB-I in the proband. A more detailed evaluation of the staining of six proteins is listed in Supplementary Table S1.

The proband’s mutation, p.G2088R, has previously been reported as the primary cause of RDEB-I. However, it is noteworthy that the previously reported mutation was compound heterozygous with a PTC mutation^4,8^. It was found in exon 75, subdomain *COL12* of the Col7 THD domain^4^.

The Col7 protein consists of three proα1 (VII) chains, each with a central collagenous THD domain flanked by a larger NH2-terminal noncollagenous domain and a smaller COOH-terminal noncollagenous domain 4. The THD domain is composed of repeating Gly-X-Y sequences that fold into a triple-helical structure in which glycine is in the center of the helix, while the other two amino acids are on the surface^4^. Interstrand interactions between the three proα1 (VII) chains in triple helices occur between glycine and either the X or Y amino acid ^9^. The hydrogen bond N-H(Gly)⋅O = C(X) is the most significant interstrand bond responsible for the stability of the collagen structure, along with other potential hydrogen bonds^9,10^. Therefore, it is believed that glycine is more important than the X and Y amino acids in terms of structural stability, so the substitution of glycine would have more devastating effects on patients^4,5^. Moreover, glycine is a neutral, hydrophobic molecule, while arginine is a basic, hydrophilic molecule. The substitution of glycine with arginine could change the triple helix’s polarity, thereby further destabilizing the Col7 structure.

Four RDEB-I patients have been identified to have glycine substitutions, all near the border of the subdomains, suggesting that those glycine substitution locations may affect the pathological phenotype^4^. However, only 6/13 (46%) glycine substitutions of RDEB-I patients were located in either the first three or last three Gly-X-Y triplets, while the rest (54%) were in the central regions of the subdomains^5^.

Immunofluorescence results showed significantly reduced Col7 protein expression in the proband sample compared to the control sample (Fig. 2). This is strong evidence that the proband suffers from the dystrophic EB subtype. The signal was weak and discontinuous. This finding correlated with the proband’s homozygous mutation in the *COL7A1* gene since we noticed a decrease in, not a total loss of, the proband’s collagen VII protein.

Type IV collagen was found above the blister and produced a normal signal, which fits the previous finding of similar IFM from a dystrophic EB patient^1^. However, reduced signal and slight discontinuities of laminin γ2 and integrin β4 were observed, corresponding to the interpretation of IFM results in patients with junctional EB^1^. This may call into question our diagnosis of RDEB-I in the proband. However, we should also consider the consequence of decreased Col7 expression in terms of the interaction between Col7 and these proteins, which are all localized to the same dermal - epidermal junctions. Moreover, the reductions in laminin γ2 and integrin β4 expression compared to the control levels were not as severe as those of Col7; thus, we suggest that experimental error and other variables should also be considered. Cytokeratin 5/6 antibodies showed slightly attenuated signals, and integrin α6 antibodies showed similar signals compared to those of the control. These antibodies are used as target proteins to diagnose basal EB simplex and junctional EB, so these results may support the hypothesis that our proband does not suffer from EB simplex or junctional EB^1^.

The previously reported cases of glycine substitution at p.G2088 are both compound heterozygous with a PTC mutation, to the best of our knowledge^4,8^. In patients with generalized RDEB - the most severe type of RDEB - homozygous PTC mutations in *COL7A1* were often found, resulting in a total lack of Col7^8^. Accordingly, heterozygous compound mutations with a PTC mutation and a codon substitution may have less severe phenotypes, resulting in milder symptoms and more localized blisters. Since our proband was homozygous for a codon substitution without any PTC mutation, it could be speculated that he should have reduced Col7 and even milder symptoms. However, he suffered from other quite serious symptoms, including nail dystrophies and enamel hypoplasia.

Since identical glycine substitution mutations caused both dominant and recessive inheritance, we are skeptical that underlying factors affect the mode of inheritance of EB^11^. Therefore, genetic evidence from our Vietnamese patient and his family may play an important role in supporting further studies of epidermolysis bullosa, because these data include all three allelic combinations of the mutation NM_000094.4: c.6262G>A, p.G2088R.

## Supplementary information

Supplementary Material

## Data Availability

The relevant data from this Data Report are hosted at the Human Genome Variation Database at 10.6084/m9.figshare.hgv.3166.
