# Correction: A rare homozygous missense mutation of COL7A1 in a Vietnamese family

**DOI:** 10.1038/s41439-022-00201-0

**Published:** 2022-06-06

**Authors:** Nguyen Thuy Duong, Luong Thi Lan Anh, Nguyen Huu Sau, Nguyen Bao Anh, Noriko Miyake, Nong Van Hai, Naomichi Matsumoto

**Affiliations:** 1 Institute of Genome Research, Vietnam Academy of Science and Technology, Hanoi, Vietnam; 2 Hanoi Medical University Hospital, Hanoi Medical University, Ministry of Health, Hanoi, Vietnam; 3 Hanoi Medical University and National Hospital of Dermatology, Ministry of Health, Hanoi, Vietnam, Hanoi, Vietnam; 4 National Research Institute, National Center for Global Health and Medicine, Tokyo, Japan; 5 Yokohama City University Graduate School of Medicine, Yokohama, Japan

**Keywords:** Genetics, Molecular biology

Correction to: *Human Genome Variation* 10.1038/s41439-022-00192-y, published online 17 May 2022

In the original version of this article, the given and family names of Noriko Miyake and Naomichi Matsumoto were incorrectly structured. The name was displayed correctly in all versions at the time of publication.

The original article has been corrected.

