# Supplementary material for: A rare homozygous missense mutation of COL7A1 in a Vietnamese family
Source: Hum Genome Var. 2022 May 17;9:13. doi: 10.1038/s41439-022-00192-y (PMC9113988; doi:10.1038/s41439-022-00192-y)
Supplement: Supplementary file 1 — Supplementary Material [file 41439_2022_192_MOESM1_ESM.docx]

Supplementary data 1. Evaluation of Figure 2’s immunofluorescence results of rubbed skin samples on proband’s forehead with corresponding controls.

| **Protein** | **Control** | **Rubbed skin sample (forehead)** |
| --- | --- | --- |
| Type IV Collagen | +++ | +++  (Above the blister) |
| Laminin γ2 | +++ | ++  (Above the blister) |
| Type VII Collagen | +++ | +  (Low, interrupted, above and under the blister) |
| Cytokeratin 5/6 | ++ | +  (Above the blister) |
| Integrin β4 | +++ | ++  (Above the blister) |
| Integrin α6 | +++ | +++  (Above the blister) |
